# Editorial: The application of green chemistry in biomass valorization: green route, green catalyst and green solvent

**DOI:** 10.3389/fchem.2023.1277256

**Published:** 2023-08-22

**Authors:** Chao Xie, Zupeng Chen, Chang Geun Yoo, Xiaojun Shen, Qidong Hou, Leslie Boudesocque-Delaye

**Affiliations:** ^1^ National and Local Joint Engineering Research Center on Biomass Resource Utilization, College of Environmental Science and Engineering, Nankai University, Tianjin, China; ^2^ Jiangsu Co-Innovation Center of Efficient Processing and Utilization of Forest Resources, International Innovation Center for Forest Chemicals and Materials, College of Chemical Engineering, Nanjing Forestry University, Nanjing, China; ^3^ Department of Chemical Engineering, College of Environmental Science and Forestry, State University of New York, Syracuse, NY, United States; ^4^ Beijing Key Laboratory of Lignocellulosic Chemistry, Beijing Forestry University, Beijing, China; ^5^ EA 7502 SIMBA, Université de Tours, Faculté de Pharmacie, Tours, France

**Keywords:** biomass transformation, biomass utilization, biomass materials, green chemistry, green solvent

## Introduction

The rapid growth of the population has resulted in the drastic exhaustion of non-renewable fossil resources and a dramatic increase in atmospheric CO_2_ concentration, which in turn led to severe energy and environmental crises. Therefore, it is highly urgent to develop renewable energy to meet the sustainable development of society. Biomass valorization presents a great approach for the substitute of fossil sources. However, the conversion of biomass into desirable products requires a series of complex deconstruction, catalytic conversion, separation, and purification processes (Hou et al., 2021), the harsh conditions may cause a series of environmental problems. Green Chemistry is defined as the “design of chemical products and processes to reduce or eliminate the use and generation of hazardous substances.” (Anastas, 2007), the application of green chemistry principles in biomass valorization is important and has become a steadily increasing field of research.

The Research Topic “*The Application of Green Chemistry in Biomass Valorization: Green Route, Green Catalyst and Green Solvent*” covers the green approach in biomass valorization, including green routes, green catalysts, and green solvents. Here we sincerely appreciate the 28 authors for their nice work on this Research Topic. Following are the highlights drawn from their contributions to this Research Topic.

## Oxidation of glucose to gluconic acid by photocatalytic strategy

Solar energy is considered a promising energy source due to its inexhaustibility, universality, high capacity, and environmental friendliness (Gong et al., 2019). The innovative combination of photocatalysis with biomass transformation represents a promising approach to achieving sustainability in chemical processes (Granone et al., 2018). The contribution by Zhang et al. designed a photocatalysis by regulating the composition and structure, they investigated the insertion of different central metal ions, Fe, Co, Mn, and Zn, into porphyrazine loading with SnO_2_ for access to the more efficient transformation of glucose into value-added organic acids in aqueous solution at mild reaction conditions. 41.2% conversion of glucose can be reached with 85.9% selectivity of organic acids (including glucaric acid, gluconic acid, and formic acid) after 3 h when using SnO_2_/CoPz as a photocatalyst. They found that the central metal in metaloporphyrazine on the surface of SnO_2_ has a significant effect on the separation of photogenerated charges, changing the adsorption and desorption of glucose and products on the catalyst surface.

## Transfer biomass-derived HMF into high-value-added chemicals by electrocatalytic strategy

Electricity is considered potential green energy as it can be generated from wind, solar, geothermal, and closed-loop biomass facilities (Menz, 2005). Besides, the electrocatalytic conversion of biomass-derived platform chemicals provides an approach to storing large-scale renewable energy in chemical bonds (Du et al., 2018). Liu et al. designed a nickel phytic acid hybrid (Ni-PA) by using natural phytic acid as a building block for the electrocatalytic oxidation of HMF to 2, 5-furan dicarboxylic acid at 1.6 V vs. RHE, the yield of 2, 5-furan dicarboxylic acid and Faraday efficiency could reach 99.1% and 90%, respectively. Detailed investigation revealed that the good catalytic performance can be attributed to the coordination of nickel ion and phosphate groups of the phytic acid molecule, the higher electrochemical surface area, and lower charge transfer resistance than other tested catalysts.

## Co-production of biohydrogen (H_2_) and biomethane (CH_4_) from biomass sources by biotechnology

The anaerobic fermentation process also presents a green and effective way to generate high value-added products from biomasses sources by biotechnology. Nawaz et al. assessed the co-production of biohydrogen (H_2_) and biomethane (CH_4_) by a two-stage anaerobic fermentation process utilizing Atriplexcrassifolia as feedstock. In the first stage, 5 g Atriplexcrassifolia and 10 mL pretreated sewage sludge were processed at 37°C and pH 5.5 after 48 h of incubation, the maximum cumulative hydrogen production, hydrogen production, hydrogen production rate was 66 ± 0.02 mL, 13.2 ± 0.03 mL/g, and 1.37 ± 0.05 mL/h, respectively. In the second stage, 40 mL of hydrogenic slurry with 80 mL inoculum was incubated at 45°C and pH 8 for 18 days, the maximum cumulative methane production, methane yield, and methane production rate can be reached at 343 ± 0.12 mL, 8.5 ± 0.07 mL/mL, and 0.8 ± 0.05 mL/h, respectively.

## The replacement of fossil sources with biomass derivates to produce high-value products

Polymer materials derived from fossil resources are widely used in our daily life. However, these polymers derived from petroleum resources are generally non-biodegradable and most of them are buried or burned after the end of their useful lives, leading to serious environmental problems. Thus, the development of biodegradable biomaterials to substitute fossil-based polymers has attracted much attention. Wang et al. reported the synthesis of glycidyl 4-pentenoate from lignocellulose-based 4-pentenoic acid, which was further copolymerized with CO_2_ using a binary catalyst SalenCoCl/PPNCl to produce bio-based polycarbonates with vinyl side chains and molecular weights up to 17.1 kg/mol. By introducing petrochemical-derived propylene oxide as the third monomer, the GPA/PO/CO_2_ terpolymer can be produced. The glass transition temperature can be adjusted from 2°C to 19°C by controlling the molar feeding ratio of GPA to PO from 7:3 to 3:7.
